# Prenatal Hypoxia in Different Periods of Embryogenesis Differentially Affects Cell Migration, Neuronal Plasticity, and Rat Behavior in Postnatal Ontogenesis

**DOI:** 10.3389/fnins.2016.00126

**Published:** 2016-03-31

**Authors:** Dmitrii S. Vasilev, Nadezhda M. Dubrovskaya, Natalia L. Tumanova, Igor A. Zhuravin

**Affiliations:** ^1^I. M. Sechenov Institute of Evolutionary Physiology and Biochemistry of the Russian Academy of SciencesSaint Petersburg, Russia; ^2^Research Center, Saint-Petersburg State Pediatric Medical UniversitySaint Petersburg, Russia

**Keywords:** prenatal hypoxia, brain development, cortex, neurogenesis, cell migration, neuronal plasticity, dendritic spines, rat behavior

## Abstract

Long-term effects of prenatal hypoxia on embryonic days E14 or E18 on the number, type and localization of cortical neurons, density of labile synaptopodin-positive dendritic spines, and parietal cortex-dependent behavioral tasks were examined in the postnatal ontogenesis of rats. An injection of 5′ethynyl-2′deoxyuridine to pregnant rats was used to label neurons generated on E14 or E18 in the fetuses. In control rat pups a majority of cells labeled on E14 were localized in the lower cortical layers V-VI while the cells labeled on E18 were mainly found in the superficial cortical layers II-III. It was shown that hypoxia both on E14 and E18 results in disruption of neuroblast generation and migration but affects different cell populations. In rat pups subjected to hypoxia on E14, the total number of labeled cells in the parietal cortex was decreased while the number of labeled neurons scattered within the superficial cortical layers was increased. In rat pups subjected to hypoxia on E18, the total number of labeled cells in the parietal cortex was also decreased but the number of scattered labeled neurons was higher in the lower cortical layers. It can be suggested that prenatal hypoxia both on E14 and E18 causes a disruption in neuroblast migration but with a different outcome. Only in rats subjected to hypoxia on E14 did we observe a reduction in the total number of pyramidal cortical neurons and the density of labile synaptopodin-positive dendritic spines in the molecular cortical layer during the first month after birth which affected development of the cortical functions. As a result, rats subjected to hypoxia on E14, but not on E18, had impaired development of the whisker-placing reaction and reduced ability to learn reaching by a forepaw. The data obtained suggest that hypoxia on E14 in the period of generation of the cells, which later differentiate into the pyramidal cortical neurons of the V-VI layers and form cortical minicolumns, affects formation of cortical cytoarchitecture, neuronal plasticity and behavior in postnatal ontogenesis which testify to cortical dysfunction. Hypoxia on E18 does not significantly affect cortical structure and parietal cortex-dependent behavioral tasks.

## Introduction

The consequences of action of pathological factors during brain embryogenesis on development of brain structure and animal or human behavior in later life depend to a great extent on the period of pregnancy when they have been applied. However, the mechanisms underlying different outcomes of pathologic conditions are not fully understood. Development of the cortical plate of rat fetuses is a good model to investigate the specific character of damage in different cell populations resulting from insults at various stages of embryogenesis. The period of radial migration of neuroblasts into the cortical plate is crucial for development of proper cortical architecture including stratification and formation of minicolumns which underlie behavioral reactions and cognitive functions in postnatal ontogenesis (Rakic, 2006; Hammond et al., 2010; Sekine et al., 2011). Earlier it was shown that neurons fail to acquire their proper position and remain scattered within inappropriate cortical layers in the fetuses when exposed to ionizing radiation (Rakic, 1988), ultrasound (Ang et al., 2006), or toxic agents (Miller, 1986; Aronne et al., 2011) during the period of radial cell migration into the cortical plate resulting in behavioral dysfunctions (Caviness and Rakic, 1978).

Hypoxia is a very common prenatal pathology, which often leads to brain malfunctioning in the postnatal period. Although the effects of oxidative stress caused by hypoxia are short-term (within a few days), hypoxia in the embryonic period leads to various structural and functional abnormalities in the postnatal period (Zhuravin, 2002; Golan and Huleihel, 2006; Vasilyev et al., 2008; Zhuravin et al., 2009). Although there are some studies of neuroblast migration under the effect of prenatal stressors the data on their long-term consequences (Rakic, 1988; Ang et al., 2006; Aronne et al., 2011) are insufficient. Prenatal hypoxia was shown to cause functional abnormalities in postnatal ontogenesis of animals (Zhuravin et al., 2004; Golan and Huleihel, 2006; Dubrovskaya and Zhuravin, 2010) but the underlying mechanisms were not sufficiently analyzed. In particular, there was no comparative evaluation of the effects of hypoxia at different stages of pregnancy on the character of neuronal migration in embryogenesis and cortical organization and functions in early postnatal life and adulthood. The aim of this work was to investigate the consequences of prenatal hypoxia on different days of gestation and cortical neurogenesis (E14 or E18) on cell generation and migration into the developing cortical plate by evaluating the number, type, and localization of cortical neurons, the density of labile synaptopodin-positive dendritic spines and behavior in the postnatal ontogenesis of rats.

## Methods

### Animals

All animal experiments were performed in accordance with the Directive #86/609 of the Council of European Communities for the protection of animals used for experimental and other scientific purposes and in accordance with the guidelines of the Russian Academy of Sciences (RAS) approved by the Scientific Council of the Institute of Evolutionary Physiology and Biochemistry of RAS, St. Petersburg, Russia.

### Cell labeling and prenatal hypoxia

Pregnant female Wistar rats (total *n* = 64) on the 14th or 18th day of gestation were injected with a DNA-replication marker 5′ethynyl-2′deoxyuridine (EdU, one intraperitoneal injection in a dose of 25 mg in 0.5 ml of saline) to label cells generated in the proliferative zone (Rakic, 1988) destined to the cortical layers V and VI (14th embryonic day—E14) or to the superficial cortical layers II-III (18th embryonic day—E18). Within the same day (1 h after injection) some females were exposed to acute normobaric hypoxia in a 100 l chamber where oxygen content was decreased from 20.7 to 7.0% and maintained at this level for 3 h. Concentration of CO_2_ in the chamber was no >0.2% and the temperature was +22°C. Another group of EdU-injected animals was kept under normal oxygen content. The offspring of all groups of animals was analyzed in further experiments on different days of postnatal ontogenesis. When calculating the age of rat pups, the day of birth was taken as P0.

### Animal behavior

To analyze the effects of prenatal hypoxia on the functional state of brain sensorimotor cortex and development of animal reactions the following specific tests have been applied.

#### Whisker placing test

This test allows quantification of the functional state of the sensorimotor cortex and is widely used for assessing the severity of the lesions of this brain region after experimental stroke or other brain pathologies (Schallert, 2006). On days P0 to P20 the rats from the control group (*n* = 13) and rats subjected to prenatal hypoxia on E14 (*n* = 14) or E18 (*n* = 16) were tested for whisker placing reaction. For this an animal was lifted by the tail and the whisker area was touched by a sharp end of a horizontally oriented pencil (Thullier et al., 1997). The forelimb placing reaction was assessed for 1 min by a four-point scale: 0–absence of reaction; 1–weak, chaotic elevation of the limb without making contact with the stick; 2–rotatory movements of the head and elevation of the limb to the support; and 3–extension of the snout in the direction of the stick with accurate and precise lifting of both forelimbs onto the support. The mean ranking was then calculated for each animal and compared in the control and both hypoxic (E14 and E18) groups.

#### Training to perform an instrumental reflex

Young (P20) or adult (P90) male rats were trained to touch or push a piston attached horizontally to the front wall of the experimental chamber with an automatic feeder and instruments for recording movements and delivery of reinforcement (Zhuravin and Bures, 1986, 1989) with some modifications (Dubrovskaya and Zhuravin, 1995; Zhuravin et al., 2002). Young rats of the control (*n* = 20), E14 (*n* = 12), and E18 (*n* = 15) groups were trained only to touch the piston while adult rats of the control (*n* = 13), E14 (*n* = 14), and E18 (*n* = 16) groups were trained to push the piston for longer than a specified period of time (usually 50 ms). Every day during two cycles of training, consisting of 64 movements each, the rats were trained to use the forelimb to touch (young rats) or to press (adult rats) the piston. Young rats were considered being able to learn the reflex well when they touched the piston during all 64 movements in the cycle. Adult rats were considered to learn the reflex well when the average time of pushing the piston in each session was ≥50 ms for 6 or 7 successive sessions (*p* < 0.05; *t*-tests). The rats whose average pushing time in a session did not significantly change during the training were considered being unable to learn the test.

### Analysis of migration and positioning OF cortical neurons generated on E14 or E18

To evaluate the position of neuronal cells generated on E14 or E18, the pups of female rats injected with EdU were decapitated on P5 when the number of labeled cells is high enough to be analyzed. The number of pups in each group is given in Table 1. The brains were extracted, fixed in 10% formalin in phosphate-buffered saline (PBS, pH 7.4), cryoprotected in 20% sucrose in PBS, frozen, and sectioned at the coronal plane. Brain slices (20 μm) were stained with Hoechst 33342 to reveal cortical stratification and to count the total number of cells. To expose the final position of the cells generated on E14 or E18 and labeled with EdU the sections were stained using the Click-iT® EdU Alexa Fluor® 488 Imaging Kit (Invitrogen, USA) following the manufacturer's protocol. In selected sections, the cells were also immunolabeled with a neuronal marker Fox3 (ab104224; Abcam, Bristol, UK; dilution 1:1000) to prove that the EdU-positive cells are neurons. To analyze the pattern of migration and positioning of E14- or E18-generated cortical neurons, the number of EdU-labeled cells within a 500 μm—wide area of the parietal cortical tissue from the layer I to VI from Bregma +0.20 mm (Paxinos and Watson, 2006) (see **Figure 2B**) was calculated. A ratio of the EdU-positive cells to total Hoechst-labeled cells was calculated in each investigated cortical slice. The mean ratio for different animal groups was compared using unpaired two-tailed Mann–Whitney *U*-tests (*p* < 0.05). The ratio of the number of EdU-labeled cells within the superficial cortical layers (the layers II-III) and lower layers (V-IV) layers was also calculated and compared in control and hypoxia-exposed pups using the unpaired two-tailed Mann–Whitney *U*-test (*p* < 0.05).

**Table 1:** **Pattern of migration and positioning of cortical neurons generated on E14 or E18**.

	**Number of pups**	**Number of cortical slices analyzed**	**Number of counted cells**	**Total number of EdU-labeled cells per 500 μm wide cortical column (mean ±SEM)**	**Number of EdU-labeled cells scattered in the layers II-III (for the labeling on E14), or in V-VI (for E18) cortical layers, presented as % to the total number of EdU-labeled cells (%)**
Control rats (EdU labeling on E14)	14	140	586460	17.5 ± 0.9	19.1 ± 5.2
Hypoxia on E14 (EdU labeling on E14)	12	120	479400	9.7 ± 0.8	42.0 ± 9.4
Control rats (EdU labeling on E18)	9	90	377460	62.8 ± 0.6	17.3 ± 7.2
Hypoxia on E18 (EdU labeling on E18)	10	100	407800	23.1 ± 0.8	28.1 ± 9.8

The mean number of EdU-labeled cells in the parietal cortex of ras subjected to hypoxia on E14 or E18 is lower than in control animals. The number of EdU-positive cells per 500 μm wide cortical column (500 μm -wide area of the cortical tissue from the layers I to VI) is presented as mean ± SEM. The mean number of EdU-labeled cells scattered in the II-III cortical layers for cell labeled on E14 or in the layers V-VI for cell labeled on E18 is given as % of total EdU-labeled cells. These cells failed to migrate to the cortical layers to which they are destined.

### Morphological analysis of cortical neurons in postnatal ontogenesis

#### Light microscopy

The rats (*n* = 8 for each group) were decapitated on P10, P20, P30, P60, and P90 and their brains were fixed in 10% formalin in PBS (pH 7.4), cryoprotected in 20% sucrose in PBS, frozen and sectioned at the coronal plane. Brain slices (20 μm) were Nissl stained and analyzed by light microscopy using an ImagerA microscope (Zeiss, Germany). Ten cortical slices (the same area of the parietal cortex as in the EdU visualization experiment) were analyzed for each animal. The area of the cell body, as well as the longest and the shortest axes with the nucleus being oriented in the section plane were measured for each cell using the “Videotest: Master Morphology 4.2” computer program (VideoTest, Russia). The cells were classified by two numerical parameters: the size (area) and shape (ratio of the longest and shortest axes passed through the cell nucleus) of the cell body using cluster analysis (Ward's method), as described in (Vasilyev et al., 2008). The size and shape of the cell body are essential parameters to differentiate the projection cells (pyramidal cells with a high axis ratio) and interneurons (with axis ratio about 1) in different cortical layers. It allows the analysis of the number of cells belonging not only to different morphotypes but also to different functional groups. The mean number of cells belonging to the different morphotype classes was counted for each cortical slice in rats exposed to hypoxia on E14 or E18 and compared to control group. The two-tailed t-student test for independent samples (*p* < 0.05) was used to compare the values obtained in control and hypoxia-exposed animals.

#### Immunohistochemistry

We have also performed an analysis of the number of cells labeled by neuronal and glial marker proteins in the same area of the parietal cortex as in the EdU visualization experiment. For this a sequence of sections (10 sections per animal with 80 μm between them) were randomly selected and used for immunolabeling. Sections were incubated for 3 h at 37°C in PBS containing 2% bovine serum albumin and 0.3% Triton X-100 (Merck, Darmstadt, Germany), and then overnight with a primary antibody at 4°C. Visualization of the glial cells with glial fibrillary acidic protein (GFAP-positive glia) was performed using a monoclonal Cy3-conjugated anti-GFAP antibody (C9205, Sigma, 1:100) and analyzing the emission of Cy3 at 550–650 nm wavelength. The number of neurons was analyzed using mouse monoclonal anti-Fox3 (= NeuN) antibody (ab104224; Abcam, Bristol, UK; dilution 1:500). After thorough rinsing, all sections were incubated for 1 h at 37°C with a FITC-conjugated secondary antibody against mouse IgG (ab7007, Abcam, 1:200) diluted in the blocking serum. The rat hepatic tissue was used as a negative control. For excitation of the signal a 488 nm wavelength He/Ar laser was used with emission being observed in the 496–537 nm wavelength range. The number of immunopositive cells with the optical density of cell bodies 300% higher than the background was compared in the parietal cortex of control and hypoxia-exposed animals using the unpaired two-tailed Mann–Whitney *U*-test (*p* < 0.05).

### Distribution of synaptopodin-positive dendritic spines in the parietal cortex

#### Immunohistochemistry

Synaptopodin is a specific marker of labile mushroom spines, which play an important role in neuronal network plasticity (Deller et al., 2003). The number of labile axon-spine inter-neuronal contacts in the molecular (Ist) layer of the parietal cortex in adult (P90) control rats as well as in animals exposed to hypoxia on E14 or E18 was analyzed using a random sampling method described in the previous section. Selected sections were incubated with a rabbit anti-synaptopodin antibody (S9567, Sigma 1:200) overnight at 4°C and then with a phycoerythrine PE-conjugated secondary antibody against rabbit IgG (ab7007, Abcam, 1:200). Some slices were double stained for synaptopodin and a postsynaptic marker protein PSD95 (mouse monoclonal ab2723 antibodies, Abcam, 1:1000, overnight) or synaptopodin and a presynaptic terminal marker protein synaptophysin (S5768, Sigma, 1:1000, overnight) and visualized by a FITC-conjugated secondary antibody against mouse IgG (ab7007, Abcam, 1:200). Rat hepatic tissue was used as a negative control. For excitation of PE and FITC a 488 nm wavelength He/Ar laser was used with emission of PE being detected at 652–690 nm and FITC at 496–537 nm wavelength. The number of synaptopodin-positive spines was calculated in a 100 × 100 μm area of the molecular layer of the parietal cortex just above the area where we performed counting of the number of neurons. The data obtained in hypoxia-exposed animals were compared with controls using the unpaired two-tailed Mann–Whitney *U*-test (*p* < 0.05).

#### Western blotting

The parietal cortical tissue sections (the same areas of the parietal cortex as described in the previous experiments) of adult (P90) control rats and of animals subjected to hypoxia on E14 or E18 were homogenized on ice in 0.5 M Tris-HCl buffer (pH 7.4) with 1% (of volume) of Triton X-100 and centrifuged at 2500 g (5 min, 4°C.) The amount of synaptopodin protein in the supernatant was analyzed by electrophoresis in 8% polyacrylamide gels in the presence of sodium dodecyl sulfate (SDS), followed by immunoblotting on polyvinylidene difluoride (PVDF) membranes. The membranes were incubated overnight at 4°C with a rabbit anti-synaptopodin antibody S9567 (Sigma 1:1000). Immunoreactivity was detected using horseradish peroxidase-conjugated secondary antibody (goat anti-rabbit IgG, ab6721, Abcam, 1:5000, 1 h) and visualized using Optiblot ECL Ultra Detect Kit (1.2 pg-2 ng) (ab133409, Abcam,). The amount of actin in the same samples was analyzed using a rabbit anti-actin antibody (A5060, Sigma, 1:5000). The amount of a postsynaptic marker protein PSD95 was analyzed using a mouse monoclonal antibody (ab2723, Abcam, 1:1000, overnight) followed by a secondary horseradish peroxidase-conjugated sheep anti-mouse IgG antibody (ab6808, Abcam, 1:5000, 1 h). The amount of an active presynaptic terminal marker protein synaptophysin was analyzed using a mouse primary antibody S5768 (Sigma, 1:1000, overnight) and visualized with the ab6808 secondary antibody. The relative intensity of immunoreactive bands on the membranes was quantified by densitometry using the “Videotest: Master Morphology 4.2” software program (VideoTest, Russia). Rat hepatic tissue was used as a negative control. A ratio of the intensity of the bands corresponding to the protein of interest (synaptopodin, PSD95 or synaptophysin) to actin was calculated for each sample and the data of hypoxia-exposed animals were compared with controls using unpaired two-tailed Mann–Whitney *U*-test (*p* < 0.05).

### Statistics

The data were analyzed using the PAST statistical software package (Hammer et al., 2001; http://palaeo-electronica.org/2001_1/past/issue1_01.htm). All data are presented as mean ± SEM. The data for hypoxia-exposed pups were compared with a control using the unpaired two-tailed *t*-student tests or Mann–Whitney *U*-test (*p* < 0.05). The Fisher's exact significance test (Mehta et al., 1984) was used in the analysis of the instrumental reflex data obtained in two animal groups.

## Results

### Animal behavior

#### Whisker placing test

**Figure 1:**
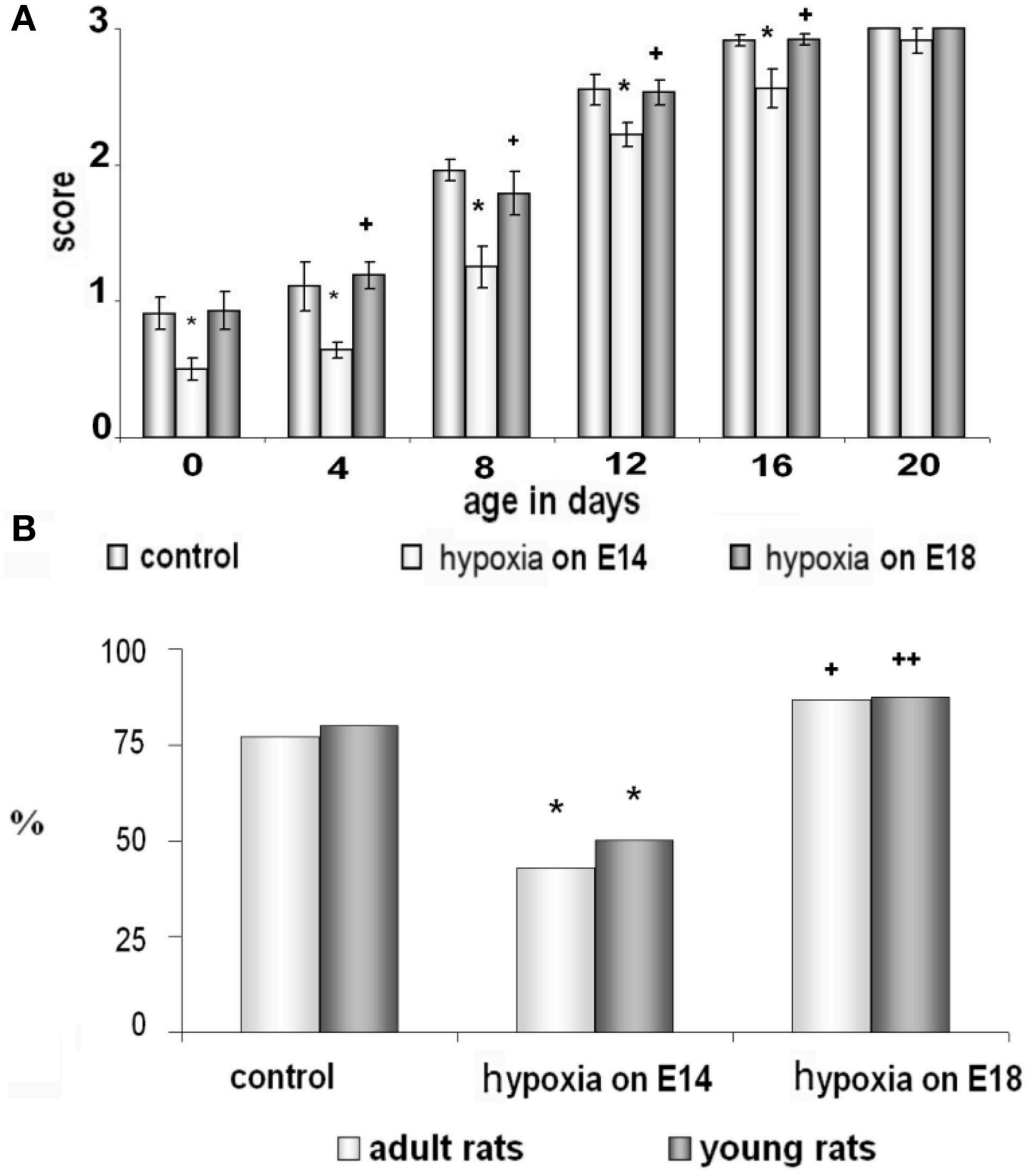
**Behavioral testing of control rats and rats subjected to prenatal hypoxia on E14 and E18. (A)** Development of the whisker placing reaction during the first month of postnatal ontogenesis on P0-P20. Data presented as the mean of whisker placing score ± SEM. Small asterisks and large asterisks—significant (*p* ≤ 0.05 and *p* ≤ 0.001, correspondingly) difference compared to control rats. Small cross and large cross—significant (*p* ≤ 0.01 and *p* ≤ 0.001, correspondingly) difference between the groups of rats exposed to hypoxia on E14 and E18. **(B)** Ability of young (dark columns) and adult (white columns) rats to learn an instrumental reflex presented as percent of animals which learn the task well from the total amount of animals taken as 100%. ^*^Significant (*p* ≤ 0.05) difference between the control rats and rats subjected to hypoxia on E14. + and ++-significant (*p* ≤ 0.05 and *p* ≤ 0.01, correspondingly) difference between two groups of rats exposed to hypoxia on E18 and on E14.

Analyzing animal motor reactions during the first postnatal month (from P0 to P20) we have observed that the pups exposed to prenatal hypoxia on E14 had a significant delay in the development of the whisker placing reaction compared to controls and pups subjected to prenatal hypoxia on E18. The animals subjected to prenatal hypoxia on E14 compared to control rats or rats exposed to hypoxia on E18 starting from P0 had a significantly lower score of forelimb placing after stimulation of their whiskers and this difference practically disappeared at P20 (Figure 1A). It indicates a disruption in development of the sensorimotor coordination caused by prenatal hypoxia on E14 but not on E18.

#### Training to perform an instrumental reflex

Using an instrumental learning paradigm, we have revealed that prenatal hypoxia on E14 resulted in reduced ability to learn a complex operant reflex (reaching or reaching with pushing by a forepaw) when tested both in young (P20) and adult (P90) animals (Figure 1B). The ability of rats to learn the task was expressed as a ratio of animals able to learn the task well to the total number of trained animals. In the group of rats, exposed to hypoxia on E18, the number of young and adult rats able to learn the reflex was not statistically different from the age-matched controls (Figure 1B). Thus, prenatal hypoxia on E14 caused significant disruption of motor behavior and learning ability of rats connected with their cortical functions, while prenatal hypoxia on E18 did not cause such changes compared to controls.

### Analysis of EdU-labeled cortical neurons generated on E14 or E18, in rat postnatal ontogenesis

#### Control group

**Figure 2:**
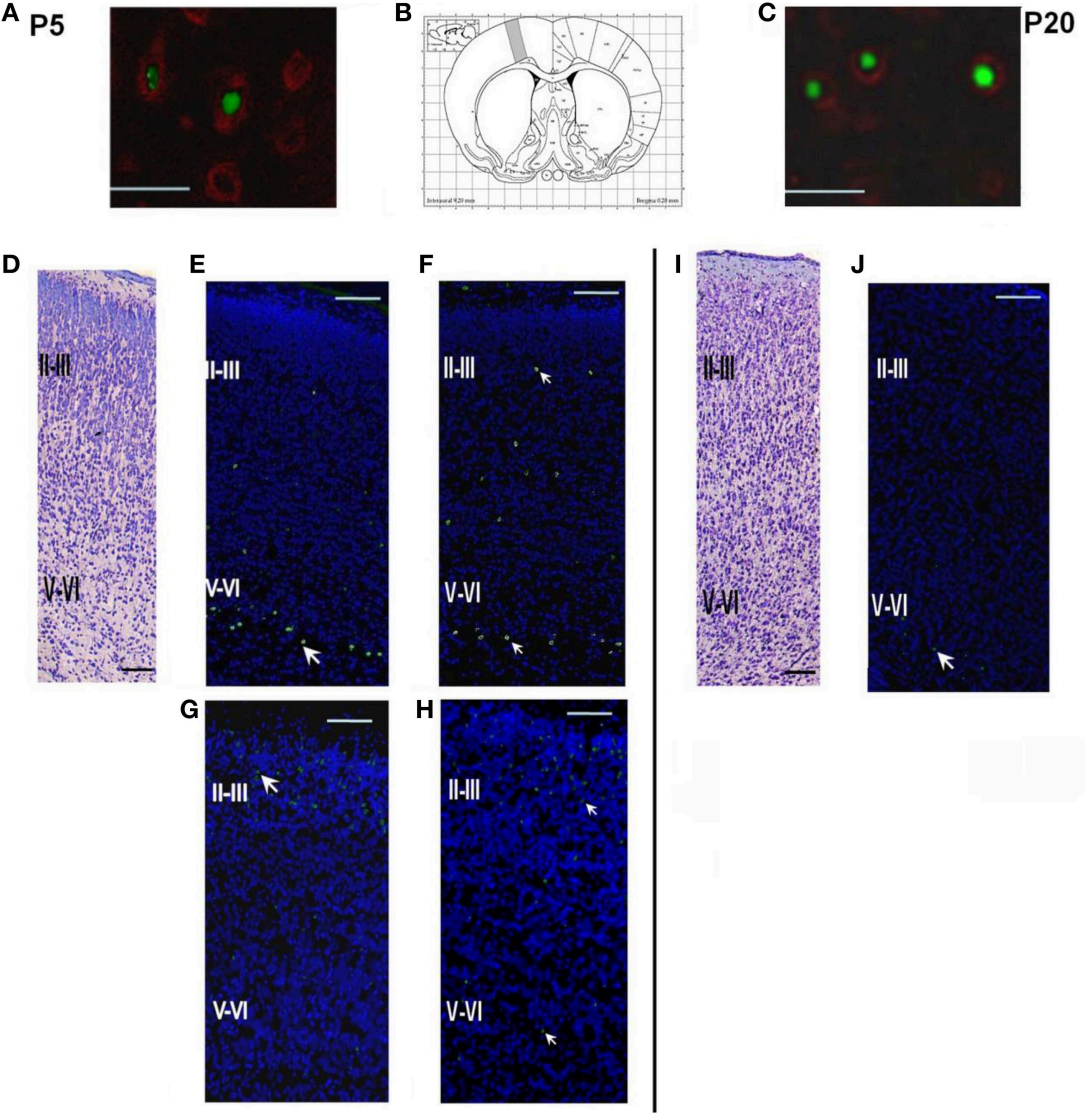
**Structure of the parietal cortex on P5 and P20 in control rats and rats subjected to prenatal hypoxia on E14 or E18. (A)** Double labeling of cortical tissue in a control rat on P5 with EdU labeling performed on E14. EdU-positive cell nuclei were stained by AlexaFluor488, Fox3-positive neurons stained by PE-conjugated secondary antibodies. Scale bar 50 μm. **(B)** Position of the analyzed areas in the parietal cortex (Bregma +0.20 mm; Paxinos and Watson, 2006). **(C)** Double labeling of cortical tissue in a control rat on P20 with EdU labeling performed on E14. EdU-positive cell nuclei were stained by AlexaFluor488, Fox3-positive neurons stained by PE-conjugated secondary antibodies. Scale bar 50 μm. **(D)** A coronal section of the cortical tissue of a control rat on P5, Nissl staining. Scale bar 200 μm. **(E)** A coronal section of the cortical tissue of a control rat on P5. EdU labeling (indicated by an arrow) on E14. The majority of the cells labeled on E14 are placed in the lower (V-VI) cortical layers. **(F)** A coronal section of the cortical tissue of a rat, exposed to hypoxia on E14, on P5. EdU labeling was performed on E14. **(G)** A coronal section of the cortical tissue of a control rat on P5 with EdU labeling performed on E18. The majority of the cells labeled on E18 are placed in the superficial cortical layers II-III. **(H)** A coronal section of the cortical tissue of a rat, exposed to hypoxia on E18, on P5. EdU labeling was performed on E18. **(I)** A coronal section of the cortical tissue of a control rat on P20, Nissl staining. Scale bar 200 μm. **(J)** A coronal section of the cortical tissue of a control rat on P20. EdU labeling performed on E14.

Double-labeling cells on P5 with EdU and a neuron-specific marker Fox3 (EdU+Fox3) showed that the majority of the cells generated on E14 or E18 in control, as well as in hypoxia-exposed animals, were neurons (Figures 2A,C). A majority of the cells EdU-labeled on E14 were localized in the lower cortical layers V-VI (Figure 2E, Table 1) while the cells labeled on E18 were mainly found in the superficial cortical layers II-III (Figure 2G and Table 1). In the course of rat development (P10-P20) the number of labeled cells decreased to 54.0 ± 8.7% compared to P5 (Figures 2D,I,J and Table 1).

#### Hypoxia on E14

Examination of brain tissue by Nissl staining on P5 has not revealed any mass cell death, changes in cell morphology, or any difference in cytoarchitecture of the cortical plate between the control and hypoxia-exposed (E14 or E18) pups. However, there was a decrease in the number of EdU-positive neurons in the parietal cortex of 5-day-old pups subjected to hypoxia on E14 (1.64 ± 0.01% of total cell number, see Table 1) compared to controls (2.93 ± 0.02% of total cell number) which suggests that prenatal hypoxia on E14 causes a decrease in neuroblast division rate. In animals subjected to hypoxia, as well as in controls, the majority of cells labeled on E14 were localized in the lower cortical layers V-VI. However, in 5-day-old pups subjected to hypoxia on E14, the number of labeled neurons scattered within the superficial layers of the parietal cortex was increased compared to controls (Figure 2F, Table 1) suggesting that prenatal hypoxia on E14 caused a failure of some neuroblasts to migrate into their proper position in the V-VI cortical layers.

#### Hypoxia on E18

On P5 in rats subjected to hypoxia on E18 we have also observed a decrease in the number of EdU-positive neurons in the parietal cortex (1.84 ± 0.02% of total cell number compared to 3.86 ± 0.01% in controls, see Table 1) suggesting that hypoxia at this stage of embryogenesis also caused a decrease in neuroblast division rate. The majority of cells labeled on E18 were localized in the superficial (II-III) cortical layers of rat parietal cortex (Figure 2H and Table 1). However, the number of EdU-positive cells scattered in the lower (V-VI) layers of the parietal cortex was higher than in controls (Table 1) suggesting that some neuroblasts failed to migrate into their proper position in the II-III cortical layers.

### Morphological analysis of cortical neurons in rat postnatal ontogenesis

#### Control rats

Analysis of cell morphology in rat parietal cortical tissue by Nissl staining on P10–P90 has been performed using such parameters as the size (area) and shape (ratio of the longest to the shortest axes) of the cell body. According to such parameters we have divided the cortical cells into four morphotypes according to DeFelipe and Fariñas (1992): 1–small non-pyramidal cells (glial cells and small non-pyramidal neurons) scattered in all cortical layers; 2–big non-pyramidal neurons (cortical interneurons) mostly localized in the layers II-III; 3–small pyramidal neurons (the ratio of the longest to the shortest axes greater than 2.5) localized in the layers II-III and V-VI of the cortex; and 4–big pyramidal cells (the ratio of the longest to the shortest axes greater than 2.5, the longest axis is more than 15 μm) mostly localized in the V-VI layer.

#### Hypoxia on E14

In pups exposed to hypoxia on E14, the number of big pyramidal neurons in the layers V-VI of the parietal cortex on P10-P20 was lower compared to age-matched controls. At later stages of postnatal development (P20-P30) we have also observed a decrease in the number of small pyramidal and non-pyramidal cells in the layers II-III of the parietal cortex of hypoxic rats (Table 2). Thus, the total number of pyramidal neurons in the layers II-III and V-VI of the cortex after prenatal hypoxia on E14 was reduced on P10-P30 by 20–30% compared to control. The number of Fox3-positive neurons was also decreased on P10-P30 (Table 2) although in older rats (P60-90) there were no differences in the number of these neurons compared to an age-matched control. At all stages analyzed we have not seen any changes in the number of GFAP-positive cells after prenatal hypoxia (Table 2).

**Table 2:** **Number of cortical neurons belonging to different morphological types in rats exposed to hypoxia on E14**.

**Type of cells**	**Days after birth**				
	**P10**	**P20**	**P30**	**P60**	**P90**
	**The number of cortical cells in rats exposed to hypoxia on E14 in % to control**				
Small non-pyramidal	101.7 ± 15.9	101.4 ± 18.7	100.8 ± 14.0	103.1 ± 15.5	110.7 ± 11.4
Big non-pyramidal	100.5 ± 9.8	81.6 ± 6.5^*^	84.3 ± 7.2^*^	102.7 ± 9.7	93.8 ± 16.3
Small pyramidal	92.8 ± 10.4	84.1 ± 7.2^*^	85.6 ± 9.8^*^	94.1 ± 15.2	105.8 ± 10.5
Big pyramidal	67.1 ± 11.3^**^	72.4 ± 8.3^**^	90.7 ± 12.9	100.6 ± 7.4	97.9 ± 15.5
Total cell number	78.6 ± 7.5^**^	59.2 ± 10.2^**^	92.9 ± 14.6	94.7 ± 8.4	97.9 ± 9.1
GFAP-positive cells	98.3 ± 5.1	105.8 ± 10.2	117.4 ± 12.5	104 ± 6.4	93.3 ± 6.5
Fox3 (NeuN)-positive	79.2 ± 6.3^*^	69.4 ± 7.1^**^	74.1 ± 5.4^*^	93.5 ± 7.7	105 ± 5.8

Neural cells were classified by their size (area) and shape (ratio of the longest to the shortest axes) of the cell body. Data are presented as the ratio (mean number of cells in rats exposed to hypoxia/mean number of cells in control rats) in % ±Standard error of the difference between the means of two samples. 100%—cell number in control rats. In each group of animals (n = 8) 10 cortical tissue areas of each animal were analyzed by Nissl staining or immunohistochemistry.

* p ≤ 0.05;

** p ≤ 0.001 using two-tailed t-student tests for independent samples.

#### Hypoxia on E18

Unlike hypoxia on E14, prenatal hypoxia on E18 caused no changes in the ratio of pyramidal to non-pyramidal cells in developing cortex and there was no reduction in the total number of pyramidal neurons on P10-P30 (Table 3).

**Table 3:** **Number of cortical neurons belonging to different morphological types in rats exposed to hypoxia on E18**.

**Type of cells**	**Days after birth**				
	**P10**	**P20**	**P30**	**P60**	**P90**
	**The number of cortical cells in rats exposed to hypoxia on E18 in % to control**				
Small non-pyramidal	97.1 ± 12.6	110.4 ± 11.2	100.0 ± 11.5	92.0 ± 10.1	115.8 ± 21.1
Big non-pyramidal	95.0 ± 15.3	95.7 ± 10.5	115.6 ± 8.8	94.5 ± 7.8	99.7 ± 17.4
Small pyramidal	104.3 ± 5.8	101.4 ± 8.2	92.4 ± 15.2	115.1 ± 19.9	92.8 ± 15.2
Big pyramidal	102.8 ± 3.7	110.6 ± 11.6	108.4 ± 7.2	100.0 ± 4.4	97.9 ± 15.5
Total cell number	100.5 ± 4.4	99.1 ± 3.8	90.7 ± 9.8	105.2 ± 9.4	105.0 ± 9.2
GFAP-positive cells	101.7 ± 3.2	104.6 ± 4.8	105.2 ± 6.7	104 ± 6.4	94.7 ± 8.9
Fox3 (NeuN)-positive	96.8 ± 8.4	108.5 ± 9.6	91.4 ± 11.5	93.5 ± 7.7	94.1 ± 10.5

Neural cells were classified by their size (area) and shape (ratio of the longest to the shortest axes) of the cell body. Data are presented as the ratio (mean number of cells in rats exposed to hypoxia/mean number of cells in control rats) in % ± Standard error of the difference between the means of two samples. 100%—cell number in control rats. In each group of animals (n = 8) 10 cortical tissue areas of each animal were analyzed by Nissl staining or immunohistochemistry. No changes (compared to control) in rats, exposed to hypoxia on E18, were observed.

### Synaptopodin distribution in the cortical tissue of adult rats (P90)

#### Control rats

**Figure 3:**
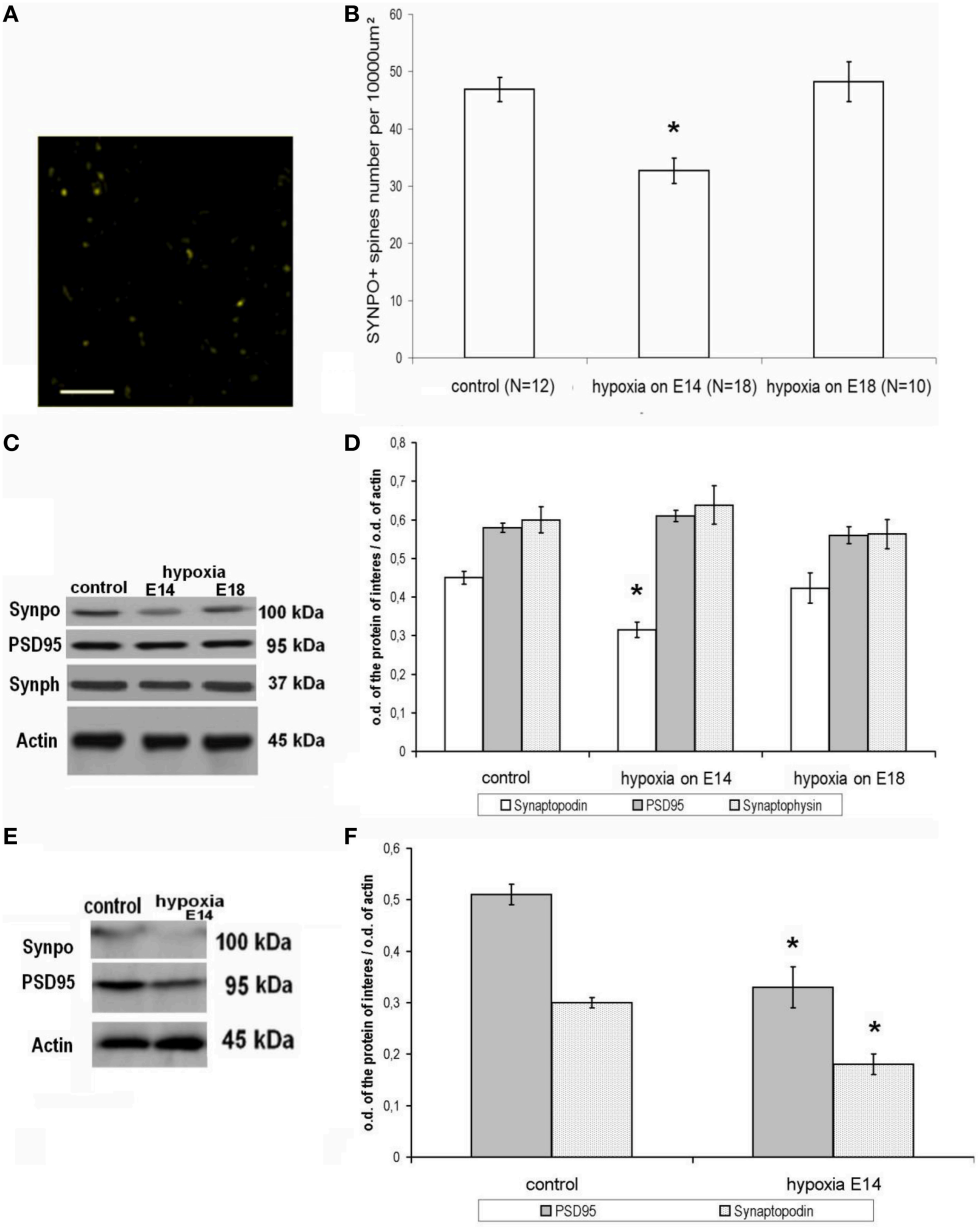
**Expression and distribution of synapse-associated proteins in rat cortical tissue on P90. (A)** Distribution of an actin-associated protein synaptopodin in the molecular layer of the parietal cortex, adult control rat. Scale: 10 μm. Indirect immunofluorescence, excitation—488 nm wavelength, emission—552–690 nm wavelength. (**B)** Number of labile synaptopodin-labeled dendritic spines in the molecular layer of the parietal cortex in adult rats (P90). Ordinate: number of synaptopodin-positive spines per 100 × 100 μm area. ^*^Statistically significant changes compared to the control (*p* ≤ 0.001). **(C)** Immunoblotting of proteins from adult rat cortical tissue with antibodies against synaptopodin (100 kDa), PSD95 (95 kDa), synaptophysin (37 kDa), and actin (45 kDa). **(D)** Ratio of the intensity of the bands corresponding to the proteins of interest (white columns—spine-associated protein synaptopodin, gray columns—postsynaptic marker protein PSD95, dotted columns—presynaptic marker synaptophysin) to the intensity of the band of actin. Data presented as the mean ratio of optical density ± SEM. ^*^Statistically significant decrease in synaptopodin expression in adult rats subjected to hypoxia on E14 compared to controls, *p* ≤ 0.05. No significant changes in PSD95 and synaptophysin can be seen *n* = 8 in each group of animals. **(E)** Electrophoretic separation of young (P30) rat cortical tissue proteins and subsequent immunoblotting against synaptopodin (100 kDa), PSD95 (95 kDa), and actin (45 kDa). **(F)** Ratio of the band intensity of the protein of interest (white, dotted columns—spine-associated protein synaptopodin, gray columns—the postsynaptic marker protein PSD95) to the actin band intensity in young (P30) rat cortical tissue. Data presented as mean optical density ratio ± SEM. Asterisk—significant difference in synaptopodin or PSD95 protein expression between control rats and rats exposed to hypoxia on E14 (two-tailed Mann–Whitney *U*-test with independent samples, *p* ≤ 0.05). No significant changes were shown in rats exposed to hypoxia on E18. *N* = 4 in each group of animals.

The pattern of distribution of the actin-associated protein synaptopodin in control adult rats on P90 is given in Figure 3A demonstrating that the majority of this protein is located in dendritic spines (immunopositive dots, 1 μm in diameter) in the molecular layer of brain cortex. On the other hand, the immunolabeled postsynaptic protein PSD95 was mostly placed near the synaptophysin-positive presynaptic terminals in different (but mostly in the molecular) cortical layers.

#### Hypoxia on E14

Prenatal hypoxia on E14 resulted in a decreased number of synaptopodin-positive spines in the molecular layer of the cortex of adult rats (Figure 3B). Moreover, hypoxia also resulted in decreased overall expression of synaptopodin protein levels compared to controls (Figures 3C,D). However, there were no changes in the amounts of the postsynaptic marker protein PSD95 and presynaptic marker protein synaptophysin (Figures 3C,D). As such we can conclude that the hypoxia on E14 resulted in a specific decrease in the number of labile synaptopodin-positive dendritic spines in the first cortical layer belonging to the apical dendrites of pyramidal neurons but did not cause significant changes in the number of PSD95-positive postsynaptic terminals and total synaptic activity. It is remarkable that in the cortical tissue of young (P30) rats exposed to hypoxia on E14 there were decreased amounts of both postsynaptic proteins synaptopodin and PSD95 (Figures 3E,F) which might lead to some abnormalities in dendritic spines formation.

#### Hypoxia on E18

In rats exposed to hypoxia on E18 we have not observed any changes either in the number of synaptopodin-positive dendritic spines in the molecular layer of the cortex (Figure 3B) or in the total synaptopodin, synaptophysin, or PSD95 content (Figure 3D).

## Discussion

Analysis of the consequences of prenatal hypoxia at different stages of embryogenesis on brain development, as well as motor and cognitive functions in animals and humans is important for understanding the underlying mechanisms of neonatal pathologies and prognosis of their outcome in later life. Investigation of behavior in rats, subjected to hypoxia on E14 or E18 performed in our study showed that prenatal hypoxia in earlier embryogenesis results in much more pronounced effect on animal motor activity and ability to learn an instrumental reflex. Using a test, which allows the analysis of the effects of various pathological factors on development of fronto-parietal neocortical regulation of motor reactions by estimating how a rat can perform forelimb placing after whisker stimulation (De Ryck et al., 1992; Fan et al., 2008), we have demonstrated that rats exposed to prenatal hypoxia on E14, but not on E18, have delayed development of this motor function in early ontogenesis. The data obtained in the operant reflex paradigm clearly demonstrated that prenatal hypoxia on E14, but not on E18, also causes a reduced ability to learn reaching movements in young and adult rats. To investigate the underlying mechanisms of the impairment caused by prenatal hypoxia on E14 compared to hypoxia on E18 we have performed detailed analysis of development of the parietal cortex in both groups of animals compared to controls.

In rat pups exposed to various harmful stimuli during embryogenesis migration of neuroblasts is reported to be compromised in different cortical areas (prefrontal, temporal, parietal, entorhinal etc.) because the mechanisms of radial migration of neuroblasts are rather common (Rakic, 1988). There are data demonstrating that hypoxia on E17 also disturbs neuroblast migration into the hippocampus (Golan et al., 2009). Continuous hypoxic conditions during brain development were reported to affect both radial (Zechel et al., 2005) and tangential (Stevens et al., 2013) migration of cortical neuroblasts causing changes in the cortical plate structure in newborn rats. However, the consequences of the failure of cell migration after prenatal hypoxia at different stages of embryogenesis on the neuronal network formation in later ontogenesis were not previously investigated. Our data suggest that prenatal hypoxia both on E14 and E18 results in a failure of some neuroblasts to migrate into their proper position in the layers V-VI or II-III of the parietal cortex, respectively. We have also found that, independently of the period when rats were subjected to prenatal hypoxia (E14 or E18), neuroblast generation rates in developing neocortex were decreased. It is worth noting that prenatal hypoxia on E14 or E18 disturbed cell generation and radial migration in a similar way and caused similar changes in final positioning of generated neurons as observed in different non-hypoxic models of prenatal pathology (Ang et al., 2006).

However, as shown in our study, hypoxia on E14 and E18 causes a selective impact on different cell populations in rat neocortex. As we noted earlier (Vasilyev et al., 2008), and confirmed in this investigation, the number of pyramidal neurons in the parietal cortex was reduced compared to the controls only in rats subjected to prenatal hypoxia on E14 but not on E18. This investigation also showed that after prenatal hypoxia on E14 the number of pyramidal neurons decreases earlier (P10-P20) in postnatal development than of non-pyramidal neurons (P20-P30). Although in adult animals subjected to prenatal hypoxia the total number of neuronal cells is normalized with aging and is not different from the controls, they still demonstrate memory deficit (Zhuravin et al., 2009). This might be a result of impairment not only in the cortex but also in the hippocampus and their neuronal networks causing long-term memory impairment (Dubrovskaya and Zhuravin, 2010; Nalivaeva et al., 2012).

Although a selective impact of prenatal hypoxia on different cell populations was suggested in the literature (Ang et al., 2006) the quantitative analysis of neurons belonging to different morphotypes in early postnatal ontogenesis after prenatal hypoxia has not been reported. The analysis of the number of cortical neurons as well as immunohistochemical evaluation of glial and neuronal marker proteins performed in this study confirmed a selective impact of prenatal hypoxia on different cell populations. Our data imply that hypoxia on E14 disturbs generation of pyramidal neurons in the lower cortical layers (V-VI) which are the first generation of cortical neurons known to be crucial for formation and function of the cortical minicolumn (DeFelipe and Fariñas, 1992). These changes in neuronal cell number and composition correlate with the deficit of motor reactions in animals observed during the first month of postnatal ontogenesis but not detected in adult animals. Since pyramidal neurons of the parietal cortex generated on E14 are known to be a part of the motor control system (DeFelipe and Fariñas, 1992) hypoxia at this period of embryogenesis leads to some dysfunctions observed as delayed development of the forepaw placing reaction. On the other hand, the decreased ability to learn reaching movements might be provoked by the structural changes underlying cortical network formation and synaptic plasticity later in postnatal life.

Development of neuronal plasticity to a great extent depends on formation of labile mushroom-shaped dendritic spines which are well-known to be capable of fast changes of their form and size resulting in modulation of synaptic transmission. One of the proteins of the spine apparatus, synaptopodin, which is regarded as a specific marker of labile mushroom spines, is important for remodeling of the spine cytoskeleton and neuronal network plasticity (Asanuma et al., 2005). There is a correlation between memory and the density and plasticity of the labile spines in the cortical areas of the brain (Deller et al., 2003). In addition to the data reported by us previously (Zhuravin et al., 2009) the results obtained in this study demonstrate that in rats subjected to prenatal hypoxia on E14 (but not on E18) in the molecular layer of the neocortex there is a decrease in the number of labile synaptopodin-positive spines, which belong to the dendrites of the pyramidal neurons, not affecting the total number of PSD95-positive postsynaptic terminals. The changes in the number of the labile spines in adult rats, exposed to prenatal hypoxia, could be provoked not only by cell death during brain development or by degeneration of the dendrite system of the cortical pyramidal neurons, but also by some specific molecular mechanism regulating spine formation. The decrease in the number of synatopodin-positive labile spines might underlie changes of neuronal network plasticity (Okubo-Suzuki et al., 2008) and testify to the impairments provoked by prenatal hypoxia.

Taking into account our data we suggest that prenatal hypoxia in the period of generation of the pyramidal neurons in the layers V-VI (E14) might affect formation of the cortical minicolumns in the postnatal ontogenesis affecting neuronal plasticity and motor functions of rats. Hypoxia in the later period of cortical neurogenesis (E18) appears not to be as crucial for neocortex formation and causes no significant changes in its structure and related specific motor functions. Further investigation of various aspects of abnormalities in brain formation caused by harmful factors acting on the organism during prenatal development can provide us with better understanding of the mechanisms of their long-term consequences and insights into pathogenesis of certain neurodegenerative disorders characteristic for human aging including dementia and Alzheimer's disease.

## Author contributions

All authors contributed equally to the experimental design and writing the paper. Dr. IZ, DV, and ND performed animals studies (hypoxia and labelleing), Dr. DV and Dr. NT have done morphology experiments, Dr. DV performed biochemical studies, Dr. ND and Dr. IZ have done behavioral experiments.

## Funding

This work was supported by Russian Foundation for Basic Research (grants numbers, 13-04-00388, 16-04-00694) and State Budget for 2013-2017 (№01201351571).

### Conflict of interest statement

The authors declare that the research was conducted in the absence of any commercial or financial relationships that could be construed as a potential conflict of interest.
